# Current progress in production of biopolymeric materials based on cellulose, cellulose nanofibers, and cellulose derivatives

**DOI:** 10.1039/c7ra11157f

**Published:** 2018-01-03

**Authors:** Hiba Shaghaleh, Xu Xu, Shifa Wang

**Affiliations:** College of Chemical Engineering, Jiangsu Provincial Key Lab for the Chemistry and Utilization of Agro-forest Biomass, Nanjing Forestry University Nanjing Jiangsu 210037 People's Republic of China xuxu200121@hotmail.com +86 25 85428369 +86 25 85428369; Jiangsu Key Lab of Biomass-based Green Fuels and Chemicals Nanjing 210037 People's Republic of China +86 25 85428369 +86 25 85428369; Jiangsu Co-Innovation Center of Efficient Processing and Utilization of Forest Resources Nanjing 210037 People's Republic of China +86 25 85428369 +86 25 85428369

## Abstract

Cellulose has attracted considerable attention as the strongest potential candidate feedstock for bio-based polymeric material production. During the past decade, significant progress in the production of biopolymers based on different cellulosic forms has been achieved. This review highlights the most recent advances and developments in the three main routes for the production of cellulose-based biopolymers, and discusses their scope and applications. The use of cellulose fibers, nanocellulose, and cellulose derivatives as fillers or matrices in biocomposite materials is an efficient biosustainable alternative for the production of high-quality polymer composites and functional polymeric materials. The use of cellulose-derived monomers (glucose and other platform chemicals) in the synthesis of sustainable biopolymers and functional polymeric materials not only provides viable replacements for most petroleum-based polymers but also enables the development of novel polymers and functional polymeric materials. The present review describes the current status of biopolymers based on various forms of cellulose and the scope of their importance and applications. Challenges, promising research trends, and methods for dealing with challenges in exploitation of the promising properties of different forms of cellulose, which are vital for the future of the global polymeric industry, are discussed. Sustainable cellulosic biopolymers have potential applications not only in the replacement of existing petroleum-based polymers but also in cellulosic functional polymeric materials for a range of applications from electrochemical and energy-storage devices to biomedical applications.

## Abstract 2

Cellulose has attracted considerable attention as the strongest potential candidate feedstock for bio-based polymeric material production.

## Graphical abstract

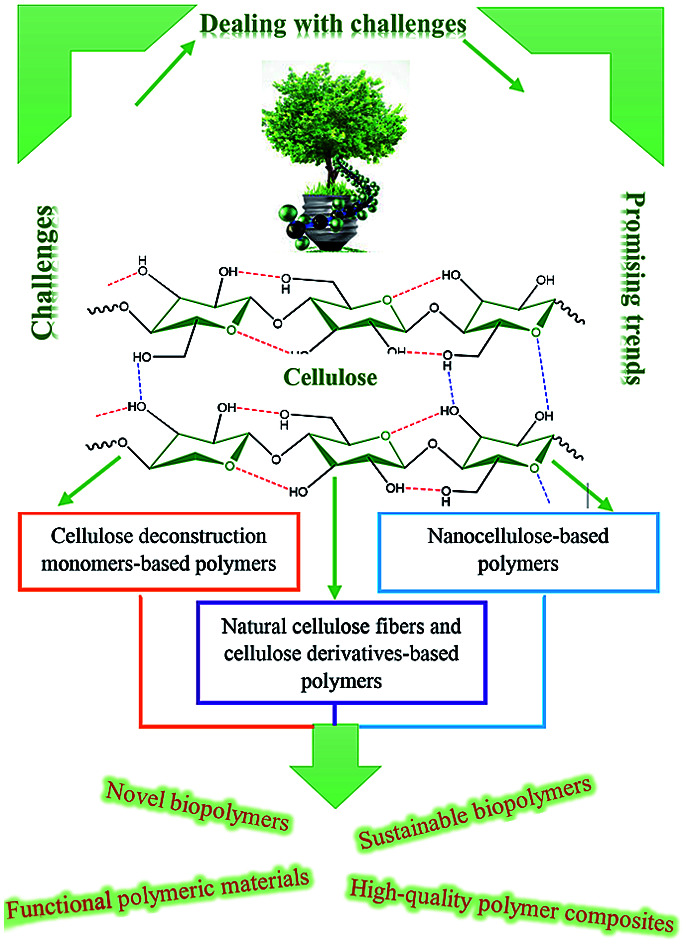

## 1. Introduction

**Fig. 1:**
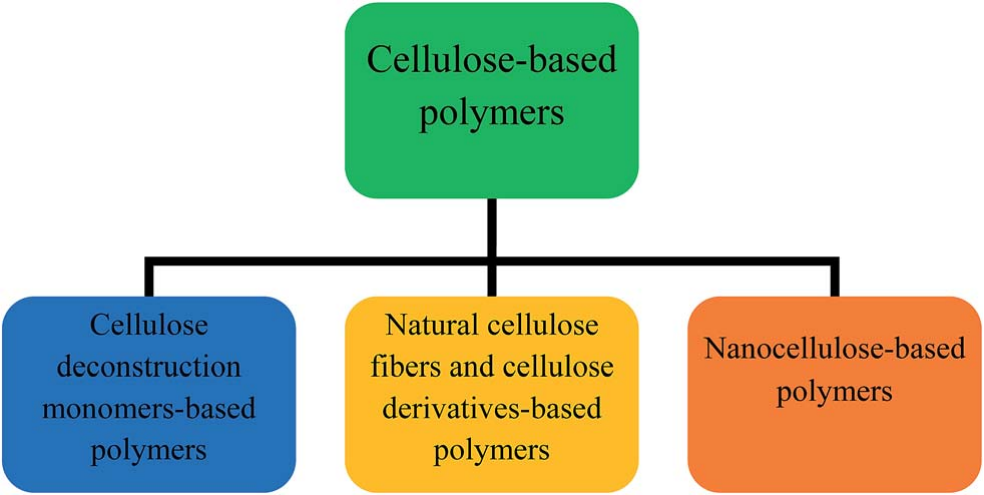
Main routes for production of biopolymeric materials from various forms of cellulose.

The development of more sustainable processes for a greener and bio-based future is a current global goal. This has led to research aimed at developing bio-based polymers to address environmental issues and to decrease the current dependence on fossil resources. In 2013, 299 million tonnes of petroleum-based polymers were produced; the average growth rate is approximately 4% per year, and demand is further increasing.^1^ It is therefore important to find alternatives to petroleum-based polymers. The production of oil-based polymers will be hindered by limited resources and rising raw material prices, and this will pave the way for the use of alternative raw materials based on renewable feedstocks. The development of biopolymer-based materials made from renewable resources is an active research area that is attracting increasing scientific and industrial attention.^2,3^ The annual bio-based share of overall polymer productions has been growing faster than overall production. It was estimated that the production capacity of bio-based polymers, which was 3.5 million tonnes in 2011, will reach nearly 12 million tonnes by 2020.^4^ The first generation of bio-based polymers was dependent on synthesizing the building blocks (monomers) from renewable resources, including lignocellulosic biomass (starch and cellulose), fatty acids, and organic waste. Worldwide production of lignocelluloses, which are the most abundant monomer resource, represent 210.7 × 10^6^ tonnes of plant material per year;^5^ this is not in competition with food supplies. Cellulose is one of the main components of lignocellulosic biomass, along with lignin and hemicellulose, and accounts for 35–50% of biomass.^6^ It is the strongest candidate for replacing petroleum-based polymers because of its abundance and eco-friendly properties such as renewability, biocompatibility, and biodegradability. Cellulose is a promising feedstock for the production of chemicals and cellulose-derived monomers. A wide variety of monomers are already obtained from cellulose *via* convenient catalytic processes^7^ and these have potential applications in production of biopolymers for use in various industries. The production capacity of cellulose-based sustainable biopolymers was 61.8% of overall bio-based structural polymer production in 2013, and production growth is increasing annually.^4^ Impressive advances in bio-based biodegradable polymeric materials based on bio(nano)polymer composites, composites films, and multifunctional composites,^8–10^ have been made in recent decades. Cellulose-based composites are the focus of a current trend toward environmentally friendly composites.^11^ Interest in the use of cellulose, cellulose nanoparticles, and cellulose derivatives as one of the (nano) composite phases has been growing in the past few years because of their excellent mechanical properties, reinforcing capabilities, low weight, low filler load requirements, biodegradability, and wide availability. They can also be used as (nano) fillers or matrices in polymer bio (nano) composites. However, the production of biopolymers from cellulose requires energetically demanding pretreatment of lignocelluloses to separate them from lignin, and subsequent biopolymer production requires deconstruction, derivatization, or nanoparticle formation. Currently, there are three main routes for the production of cellulose-based polymers (Fig. 1). The appropriate route depends on the form of cellulose being used. Different approaches to biopolymeric material production *via* these routes have been used. Unmodified cellulose fibers, chemically modified cellulose such as cellulose derivatives, and nanocellulose have been processed to give many types of biodegradable polymeric materials such as films, composites, nanocomposite systems, and composites films for various applications. Cellulose monomers have also been used for to synthesize sustainable biopolymers and functional polymeric materials. Various reviews have intermittently addressed cellulose use in biopolymeric applications. While intentionally avoids discussion of the classification of, and an introduction to, all current potential cellulose-based biopolymers. The present review covers the main production routes to cellulose-based polymeric materials and highlights the most important current achievements in this field. This review paper is divided into three sections with regard to current trends. The first section gives a critical analysis of the current status of the production of polymeric materials based on cellulose-derived monomers. The next section briefly discusses recent progress in the use of unmodified cellulose fibers and their derivatives in biopolymeric films and in biopolymer composites as fillers or bulk polymeric matrices. The last section reviews progress to date in the use of nanoscale cellulose in polymer nanocomposite materials. The last section also discusses the efficiency of nanocellulose as a reinforcing phase in various polymer nanocomposites, and the potential of nanocellulose as an ideal polymer matrix phase in nanocomposites, and their advanced applications. All three sections highlight the technological challenges and most promising applications of the corresponding cellulosic forms in polymeric material production, together with potential solutions to problems. This review will enable a better understanding of the current status of different types of biopolymers obtained by various cellulose production routes for different applications. The introduction of such sustainable solutions will develop commercial interest in cellulose as a promising biopolymer source, and help to find the most appropriate applications in this field. These potential solutions will encourage the replacement of existing petroleum-based polymers by sustainable biopolymers, and the use of cellulosic functional polymeric materials for a range of applications to meet the demands of a sustainable society.

## 2. Structures and properties of cellulosic forms

**Fig. 2:**
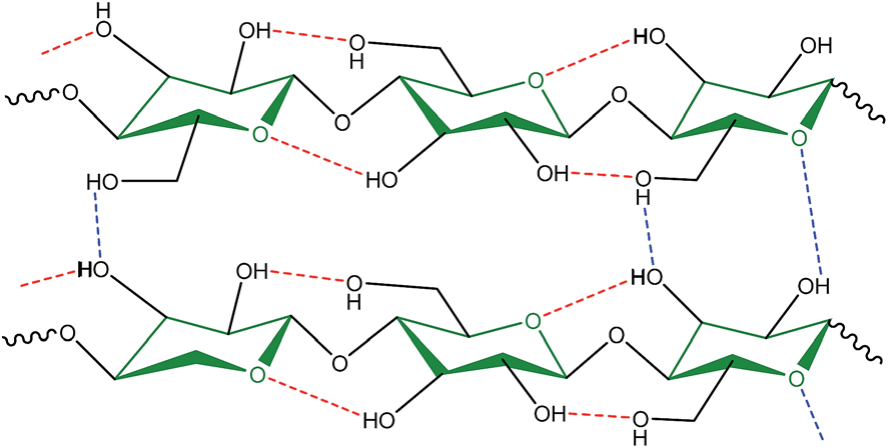
Intra- and inter-molecular hydrogen bonds in molecular structure of cellulose. Anhydroglucose units are linked by 1,4-β-glycosidic bonds.

Cellulose is the most abundant biopolymer and an important structural component of the cell walls of plants. It is also present in a wide variety of other living species such as algae, fungi, bacteria, and in some sea animals such as tunicates.^12^ Cellulose is a versatile starting material for subsequent chemical transformations because of its unique structure, which significantly affects its chemical reactions. Cellulose is a linear syndiotactic homopolymer composed of d-anhydroglucopyranose units, which are linked by β-(1→4)-glycosidic bonds.^13^ Unlike the glucose in other glucan polymers, the repeating unit of this natural polymer is a dimer of glucose, known as cellobiose.^14^ These linear glucose chains aggregate to form strong microfibrils.^15^ Many of the properties of cellulose depend on the degree of polymerization, which can vary depending on the cellulose source. Because of the high number of hydroxyl groups on the glucose rings along the skeleton, there is extensive hydrogen bonding between individual cellulose chains (intra- and inter-molecular bonds) (Fig. 2). This results in the crystallization of multiple cellulose chains into insoluble microfibrils and two structural regions, *i.e.*, crystalline and amorphous regions; this gives cellulose its high strength, stiffness, durability, and biocompatibility.^16^ The presence of three hydroxyl groups in each monomeric unit and their high reactivity gives cellulose properties such as hydrophilicity, chirality, and biodegradability.^17^ These hydroxyl groups also facilitate the chemical modification of cellulose to give cellulose derivatives *via* reaction of some, or all, of the hydroxyl groups on the repeating units in cellulose. This is possible because the abundant hydroxyl groups are suitable for chemical functionalization, *e.g.*, by etherification, carboxymethylation, cyanoethylation, and hydroxypropylation. The obtained cellulose derivatives may be more useful than cellulose because of the altered properties. Cellulose derivatives can be grouped according to their production method and substituents, *e.g.*, ester-cellulose acetate through esterification and ether-methylcellulose/carboxymethyl cellulose *via* etherification.^18^

Cellulose-derived monomers are obtained by chemical treatment of cellulose. This form can be obtained by deconstruction of cellulose into glucose, which can then be converted to a wide range of value-added chemicals such as lactic acid (LA), levulinic acid (LevA), sorbitol, and 5-hydroxymethylfurfural (5-HMF), which are important bio-based polymer platforms.

Nanoscale cellulose fibers can be isolated from a range of cellulose sources by various isolation methods, and used for production of polymeric biomaterials. Two general types of nanocellulose can be produced from cellulosic biomass *via* a top-down approach, namely cellulose nanocrystals (CNCs) and cellulose nanofibers (CNFs). CNCs and CNFs differ in terms of their production processes and structures, and can generally be separated from a given cellulose source by mechanical, chemical, a combination of mechanical and chemical, or enzymatic processes.^19^ CNCs have Young's moduli of 120 to 170 GPa, high specific surface areas (150 to 170 m^2^ g^−1^), high crystallinities (54–90%), thermal stabilities up to approximately 300 °C, lyotropic liquid crystalline behaviors, and shear thinning rheologies in CNC suspensions.^20^ Moreover, CNCs have reactive surfaces because of the abundant exposed hydroxyl groups. Such surfaces can be readily modified to give different surface properties to adjust the dispersion of a wide range of suspensions and matrix polymers, and control the interfacial properties in composites.^21^ The aspect ratios of CNFs are higher than those of CNCs; both contain crystalline and amorphous regions. Another type of nanocellulose, bacterial cellulose (BC), can be produced by bacterial processes *via* a bottom-up approach. BC has a higher purity and a higher degree of crystallinity (60–90%) than plant cellulose, and forms characteristic ribbon-like microfibrils, which give BC outstanding mechanical strength.^17,22^ Cellulose nanoparticles have a wide range of potential applications because of their low density, biodegradability, high aspect ratio, high strength and stiffness, outstanding reinforcing properties, and transparency.^23^

## 3. History of biopolymeric materials based on various forms of cellulose

Polymeric materials based on cellulose have been used in numerous fields for a long time ago. The use of cellulose originally depended on the first and second routes (Fig. 1) to give cellulosic polymeric materials based on cellulose fibers, cellulose derivatives, and cellulose-derived monomers. Cellulose was initially converted to a regenerated form, by derivatization without depolymerization, *via* the viscose process for the production of cellophane and rayon, which has been known for decades. Later, the cuoxam and lyocell methods were used instead of derivatization to synthesize cellulose polymeric products. Alternatively, cellulose can be catalytically depolymerized to the monomeric glucose form, which is the starting material for production of a huge variety of monomers by catalytic or biotechnological conversion. These cellulose-derived monomers have been used for sustainable biopolymer production.^7^ The use of cellulose derivatives as thermoplastic bulk materials was first industrialized in the 19th century. The traditional viscose route, which generates hazardous by-products (CS_2_ and H_2_S), was used to produce regenerated cellulose-based bioplastic films. Polymers based on cellulose derivatives, produced by chemical modification of cellulose, have also been used in various applications (*e.g.*, films and coatings). The main cellulose derivatives used industrially are cellulose acetate, cellulose esters for molding, extrusion, and films, regenerated cellulose for fibers, and cellulose ethers, which are widely used in construction materials, food, personal care products, paints, and pharmaceutical applications.^24^ However, the processability and use of this form of cellulose in biodegradable plastic films were limited by the lack of suitable solvents until solvent systems for dissolving cellulose became available.^25,26^ Recently, new and improved polymeric thermoplastic film materials, and functional polymeric materials such as composites and composite films have been used in industry.^27,28^

The next generation of cellulosic biopolymeric materials emerged from the third production route, and includes nanoscale cellulose fibers. This generation of cellulosic biopolymeric materials was obtained by integration of nanocellulose into various polymeric materials. Polymer composites and functional polymeric materials and composites are typical of this generation of cellulosic biopolymeric materials. Such cellulosic biopolymeric materials have attracted increasing attention because of their numerous applications in a wide range of fields. The use of cellulose in biocomposite materials has been the subject of international research since at least the mid-1980s,^29^ but has been limited to the use of cellulose fibers and regenerated cellulose as reinforcing phases in polymer composites, *e.g.*, cellulose fibers have been used as the reinforcing phase in polypropylene (PP) composites.^30^ Recently, cellulose fibers, cellulose derivatives, and nanocellulose have been used in composites and nanocomposites as matrices and fillers. Each functional role of cellulose in composites has specific applications. Research from the 1980s to the present has led to numerous new cellulose polymer composites, which are among the most important and interesting biocomposite materials. Also, numerous advanced functional polymeric materials based on cellulose have been obtained. These polymeric materials have been improved by the inclusion of glycopolymers, obtained from either glucose monomer derived from cellulose or various cellulosic forms, which act as matrices.

## 4. Sustainable biopolymers based on cellulose-derived monomer platforms in first route for cellulosic biopolymer production

**Fig. 3:**
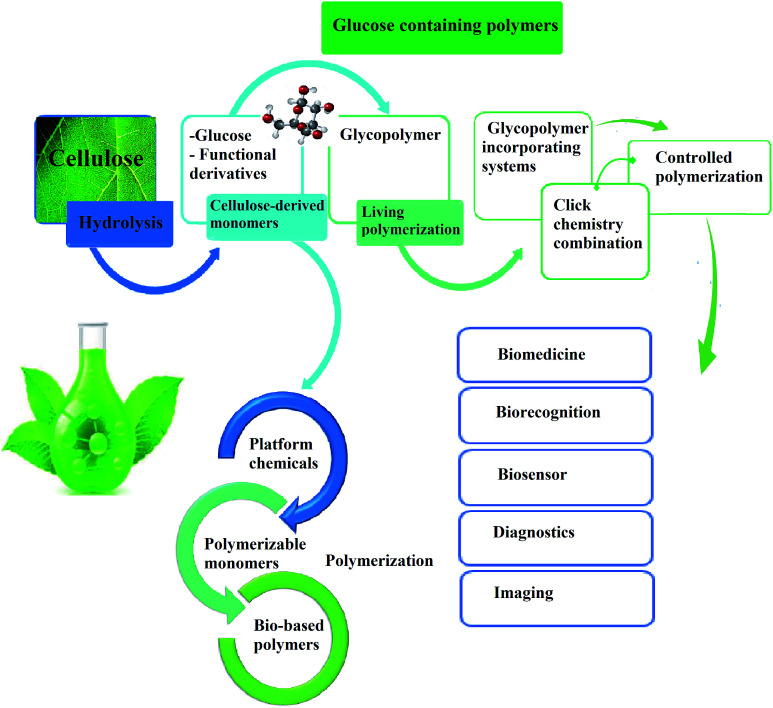
Schematic diagram of integrated routes to potential cellulose-based monomers for sustainable polymer production.

Cellulose can be depolymerized to glucose through enzymatic hydrolysis of the β-1,4-glycosidic bond^31^ or *via* catalyst-free hydrolysis in supercritical water.^32^ Recently, cellulose has been depolymerized by homogeneous or heterogeneous reactions with acids.^6,33^ Such reactions open up a wide range of new possibilities for obtaining bulk cellulose-derived monomers for polymer production. Fig. 3 shows selected routes for cellulose transformation to monomers that have high potential as future feedstocks for polymer production.

Cellulose can act as a major feedstock for sugar-containing polymers and sugar-containing polymer systems by providing a C6 (glucose) monosaccharide and its functionalized derivatives. Glucose and its derivatives can either be incorporated into a polymer backbone as a multifunctional building block to develop sugar-linked polymers, or be used as pendant groups to conjugate sugar moieties to produce polymer scaffolds for glycopolymer generation.^34^ Synthetic glycopolymers with pendant sugar moieties along the polymer backbone, or at the chain ends, can interact with lectins through multivalent interactions, mimicking the natural structures and functions of glycoproteins, therefore this type of polymer is of special interest.^35^ The multivalent properties of glycopolymers with large numbers of repeating carbohydrate units give a “cluster glycoside effect”, which greatly enhances carbohydrate–lectin binding.^36^ Advances in glycopolymer technology have enabled the design of various glycopolymer structures by living polymerization of glycopolymers (Fig. 3). The successful synthesis of such polymers by various techniques^37^ has enabled the development of well-defined glycopolymers with a variety of compositions and topologies, including homopolymers, and statistical and block copolymers. Glycopolymeric materials have great versatility and are used in many advanced biomedical and biological applications.^38^ Glucose monosaccharides are also used as the starting material for catalytic or biotechnological conversion to a large variety of fine chemicals and potential monomers such as methanol, LA, LevA, sorbitol, 5-HMF, and several dicarboxylic acids containing different numbers of carbons. Direct catalytic conversion of cellulose to platform chemicals *via* various methods, *e.g.*, using ionic liquids (ILs), has been used successfully for the challenging transformation of cellulose into renewable platform chemicals and for the separation of cellulose from the other components of lignocellulosic biomass. These cellulose-derived monomers can be used directly or further converted to polymerizable monomers for sustainable novel polymer and copolymer synthesis.^7,39^ The sustainable polymers that can potentially be obtained from cellulose-derived monomers are summarized in Table 1. Cellulose is the best candidate for ethanol production. After enzymatic or acid-catalyzed hydrolysis of cellulose, the obtained glucose is converted to ethanol *via* fermentation.^40^ Sorbitol or sugar alcohols can also be produced from cellulose *via* chemocatalytic transformations of glucose, including hydrogenation or direct hydrogenolysis.^41^ Further products are available *via* dehydration hydrogenolysis or hydrodeoxygenation reactions of sorbitol to yield isosorbide, glycerol, propylene, or ethylene glycol.

**Table 1:** Transformation of cellulose-derived platform chemicals to selected polymerizable monomers, and corresponding renewable polymers

Cellulose-based monomer	Polymerizable monomer derived	Corresponding polymers	Reference
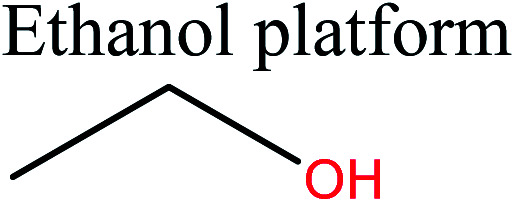	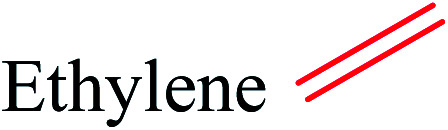	Polyethylene, polyethylene oxide polyvinyl chloride, polystyrene, polypropylene	42
	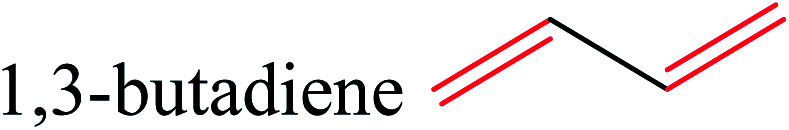	(Copolymers), polybutadiene, acrylonitrile–butadiene–styrene, acrylonitrile–butadiene styrene–butadiene	7
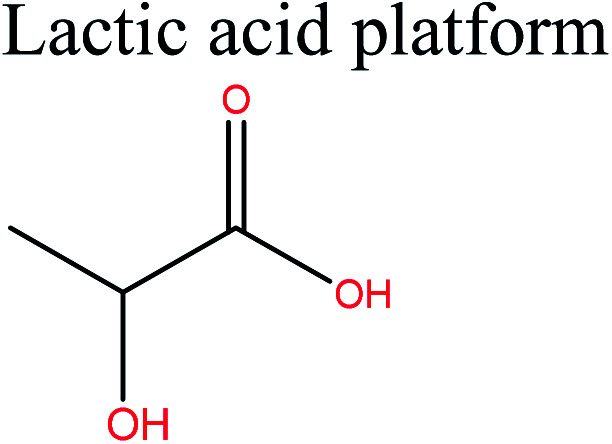	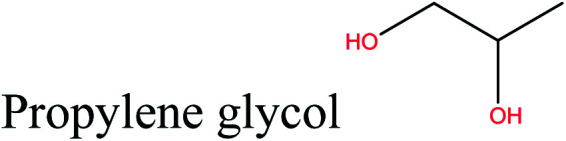	Polyester, polycarbonates, polyurethanes, polypropylene oxide	43 and 44
	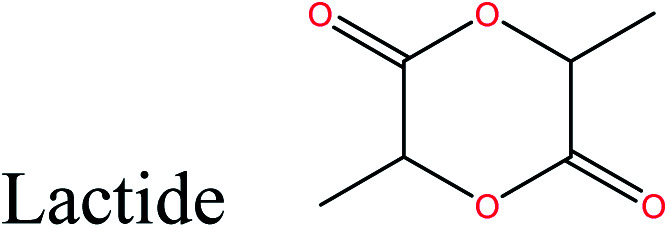	Poly(lactic acid) and its multifunctional polymeric (nano)composites and blends such PLA–PHB systems, composites and nanocomposites	45–48
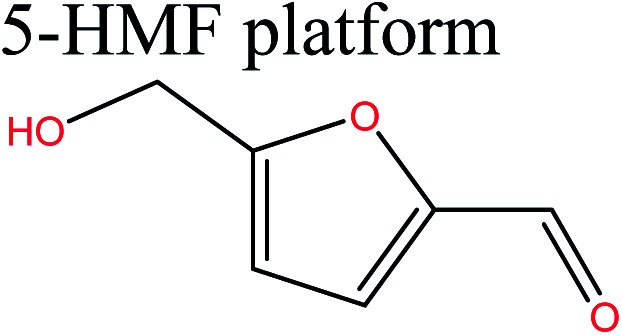	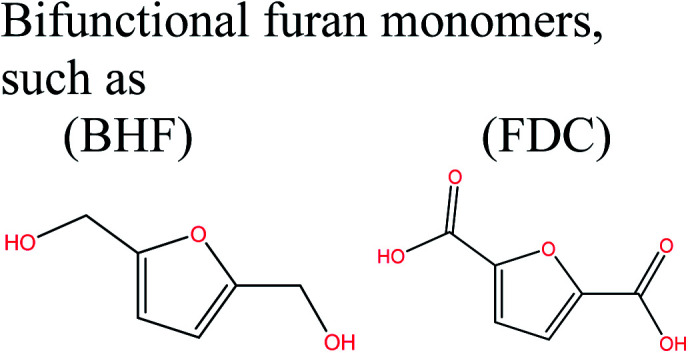	Polyurethanes, polyamides, new polyesters, (PEF), (PPF)^a^ or other polycondensates furan-based polymers	42 and 49
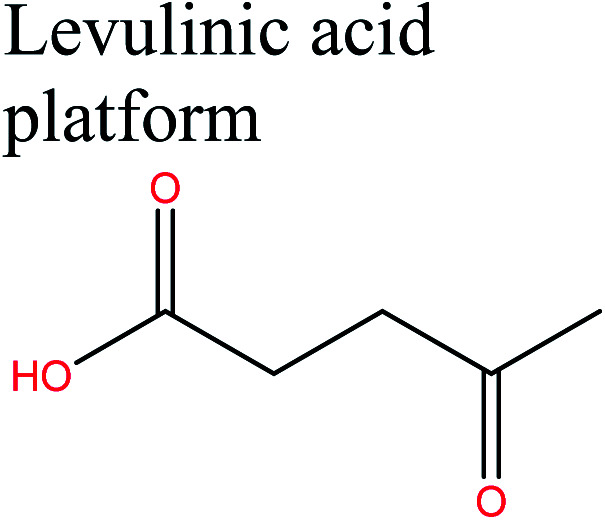	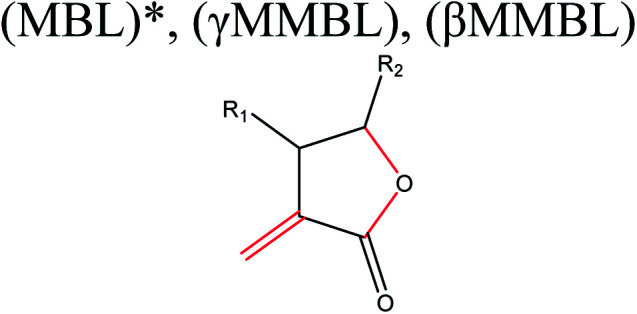	Sustainable methylene butyrolactone polymers	42
	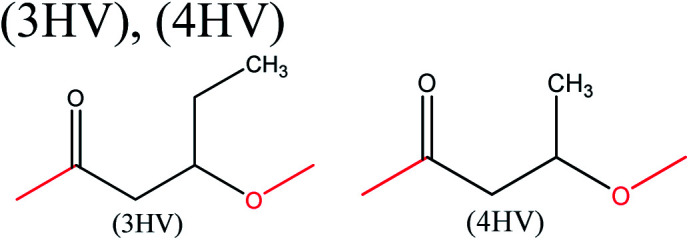	Biopolyesters (polyhydroxyalkanoates) (PHA) and their copolymers such as poly(3-hydroxybutyrate-*co*-3-hydroxyvalerate) (PHBV) and composites	50 and 51
	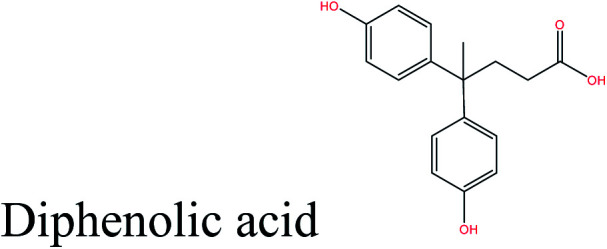	Polycarbonates	5 and 52
	LA ketals	Polyurethanes and thermoplastics	53
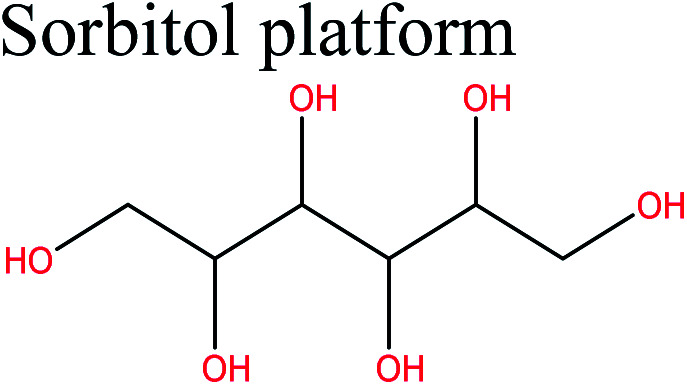	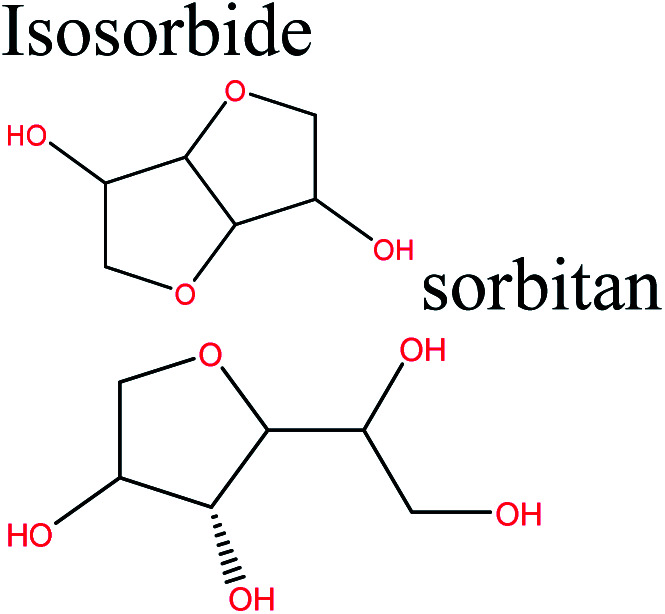	Polyesters, polyamides, polycarbonates, copolyesters, polyurethanes, polyethene isosorbide terephthalate	42
	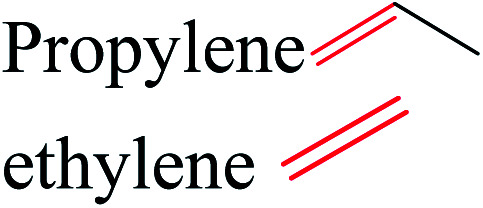	Polyethene and polypropylene	54
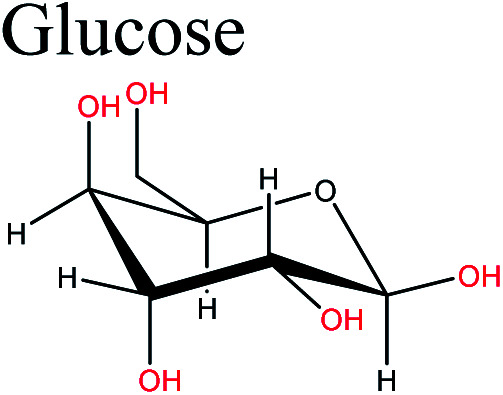	Glycopolymers	Glycopolymers, glycopolymers incorporating systems	39
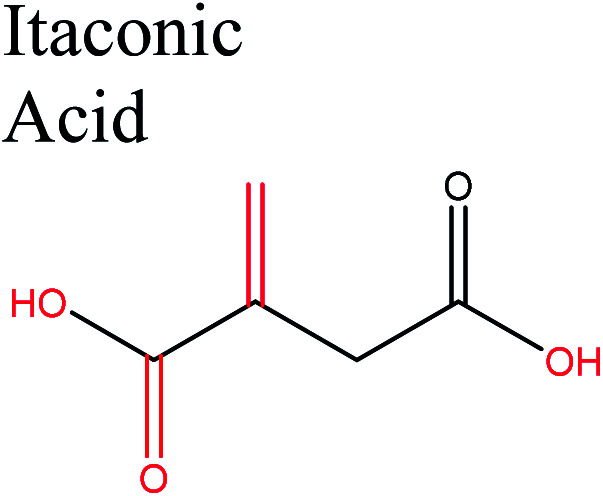	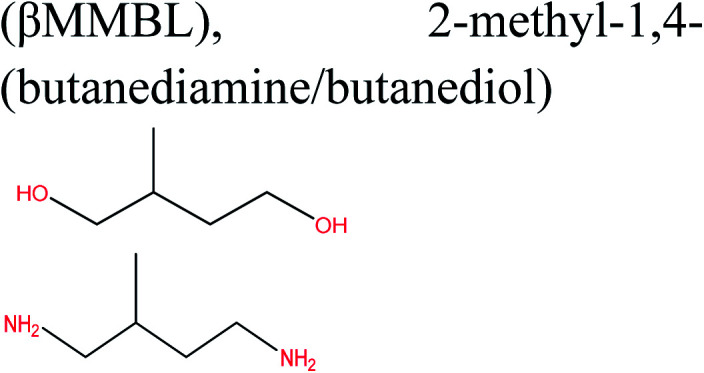	Sustainable methylene butyrolactone polymers, new polyesters, polyamides	7
	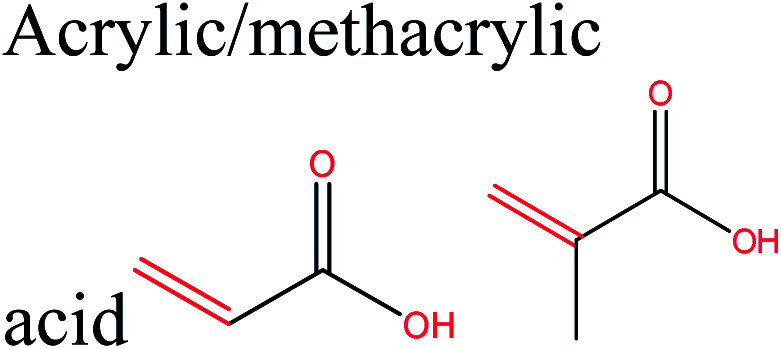	Polyacrylic acid, polymethylmethacrylate	55

a MBL: methylene-γ-butyrolactone, where R_1_ = R_2_ = H; γMMBL: γ-methyl-α-methylene-γ-butyrolactone, where R_1_ = H, R_2_ = Me; βMMBL: β-methyl-α-methylene-γ-butyrolactone, where R_1_ = Me, R_2_ = H; PEF: poly(ethylene 2,5-furandicarboxylate); PPF: poly(propylene 2,5-furandicarboxylate); FDC: 2,5-furan dicarboxylic acid; BHF: 2,5-bis(hydroxymethyl)furan; 3HV: 3-hydroxyvalerate; 4HV: 4-hydroxyvalerate. The important bonds and functional groups for derivatization and polymerization are marked.

LA is a high-potential and versatile cellulosic biomass-derived platform chemical.^56^ Currently, LA is commercially produced by fermentation of sugars^57^ at high concentrations, using a novel homofermentative, facultative anaerobe *Enterococcus faecalis* CBRD01. Glucose yields up to 98% high-purity LA. LA can serve as an active precursor and renewable feedstock for the production of a wide range of polymers *via* two routes. The first route involves dehydration, reduction, and oxidation of LA to polymerizable intermediates,^56^ which can be used to synthesize the corresponding polymers.

The second route involves direct polycondensation of LA or ring-opening polymerization of lactide, to give poly(lactic acid) (PLA).^45^ PLA is the most extensively researched and used biodegradable and renewable thermoplastic polyester, and could potentially replace conventional petrochemical-based polymers. PLA, which has a production capacity of 195 000 tonnes,^4^ has been used as an alternative to certain petroleum-based plastics in commercial applications^58^ because its mechanical properties are similar to those of poly(ethylene terephthalate) (PET) and PP. However, some of its properties, such as brittleness and a low heat distortion temperature, have restricted its use in a wide range of applications. Recent developments in the preparation of PLA blends, composites, and nanocomposites have successfully overcome the problem of brittleness and widened the range of PLA application.^46^ Fully biobased PLA-PHB/PHBV blends materials show highly promising perspectives for the replacement of traditional petrochemical-based polymers currently used for food packaging. Incorporation of high crystalline PHB to PLA matrix by melt blending is a way to increase PLA crystallinity due to its nucleation effect. Also this incorporation lead to materials with higher barrier performance and better mechanical resistance for high potential application in food packaging.^59,60^

Acid-catalyzed dehydration of glucose gives access to 5-HMF; this is the starting material for the synthesis of furan-based monomers, and could provide new substitutes for commodity polymers,^61^ similarly to LevA. Various companies have produced novel biopolymers based on LevA or its derivatives with the aim of producing cost-competitive and sustainable polymers. Most importantly, LevA can be used as the active precursor of 3-hydroxyvalerate (3HV) and 4-hydroxyvalerate (4HV) monomers, which are constituents of biosynthetic polyhydroxyalkanoates (PHAs) and their copolymers, blends, and composites produced by bacteria.^51^ Itaconic acid, which is a C5 unsaturated dicarboxylic acid, is another attractive cellulose-based monomer. It is produced by fermentation of cellulose-derived glucose by fungi, and has a global production of about 15 kilotonnes per annum. This cellulose-based monomer provides novel polymers such as poly(itaconic acid)-based polyesters and polyamides.

Notably, cellulose deconstruction to various monomers provides various petroleum-derived traditional polymers such as polyethylene (PE), polyesters, and PP. These polyolefins have exactly the same chemical, physical, and mechanical properties as their petrochemical counterparts. However, the production of these biopolymers is limited by three technological challenges: (1) isolation of cellulose from lignocellulose; (2) cellulose hydrolysis to glucose, and (3) cellulose conversion to polymerizable monomers. This situation offers both challenges and promises for the development of biomass biorefinery of cellulose from lignocellulosic biomass. For example, biopolyester production from cellulose-based ethylene monomers involves a time-consuming biological fermentation process. In addition, a number of steps are required for further ethylene and PE production. Bioethanol obtained by fermentation needs to be distilled to remove water and to yield an azeotropic mixture of hydrous ethanol, and then the ethanol must be dehydrated at high temperatures over a solid catalyst to produce ethylene and subsequently PE. Multistep biorefinery processes have been unattractive because of low oil prices and the limitations of biotechnological process. However, biopolyolefins and other polymers based on cellulosic monomers are now being manufactured for various reasons. These reasons include increasing oil prices and the environmental costs of oil-based polymers, in addition to progress in biotechnological processes and in lignocellulose pretreatment to achieve maximum cellulose extraction, such as recently reported combined pretreatment methods.^62^ However, it is necessary to make these multistep processes faster and more cost-effective. New functional catalysts such as (functionalized) ILs and combinations of different green and functional solvents (*e.g.*, IL/IL, water/IL, supercritical CO_2_/IL) are emerging. These functional catalysts give excellent one-pass catalytic processes in the presence of multifunctional catalysts for cellulose conversion to its monomers. In this context, in the development of these systems, the costs of (functionalized) ILs, especially for large-scale applications, need to be considered to make biorefining economically viable. Deconstruction of cellulose also produces cellulose-derived monomer-based polymers that have similar properties to those of the polymers obtained from crude oil, such as 2,5-furan dicarboxylic acid-based polymers like poly(ethylene 2,5-furandicarboxylate) or poly(propylene 2,5-furandicarboxylate); these are analogous to PET, which is the most common thermoplastic polymer in the world.^63^ Cellulose-derived LevA monomers provide an inexpensive and renewable carbon source for 3HV-related precursors for high-concentration PHA production, particularly poly(3-hydroxybutyrate-*co*-3-hydroxyvalerate) (PHBV) copolyesters.^64–66^ It can be converted by microorganisms into 4HV and 3HV,^67^ which enable growth and accumulation of PHA, and consider as one of the main constituents of biosynthetic (PHBV) copolyesters.^50^ PHAs are biodegradable thermoplastics with great potential as replacements for petroleum-based plastics. This is because of their physical and technological properties, which are analogous to those of some petrochemical-based thermoplastics, *e.g.*, PE and PP.^68^ The use of cellulose-based LevA as a monomer could therefore reduce the production costs of PHA because the price of the carbon source used in the fermentation represents a major percentage of the final cost. The use of a feed containing 1 g L^−1^ LevA increases PHBV copolyester production by 100%.^69^ Furthermore, control of the composition of the PHA copolyester by changing the 3HV content in poly(3HB-*co*-3HV) is desirable in industrial terms because it enables the production of thermoplastics with various degrees of flexibility and toughness, and enhanced material qualities.^70,71^ The use of cellulose-based LevA as a renewable and inexpensive feedstock could therefore facilitate the commercialization of these eco-friendly biopolyesters and their PHBV copolymers. Cellulose deconstruction also enables the synthesis of novel polymers such as poly(itaconic acid), PLA, and new polyesters based on 2,5-bis (hydroxymethyl) furan and 2-methyl-1,4-butanediamine. Cellulose deconstruction also opens up new routes for developing novel polymeric functional materials from glucose-derived monomers and their derivatives. These can be obtained without any competition with food-glucose supplies. However, the development of feasible and economic manufacturing processes for such bio-based polymers is a challenge. For these cellulose-based polymeric materials to be able to meet economic, environmental, and social needs, all the synthetic steps must be efficient, as of depolymerization of cellulose to glucose, which significantly correlates with lignocellulose pretreatment to improve cellulose isolation. However, when the goal is the production of sustainable biopolymers based on polymerizable monomers, biopolymer production is more economically viable if the direct catalytic conversion of cellulose to monomers is used during synthesis. Reactive blends, composites, and nanocomposites are constantly being used to develop new bio-based materials.

## 5. Current status of biopolymeric materials based on natural cellulose fibers and cellulose derivatives: second route for cellulosic biopolymer production

The current global current interest in the use of cellulose fibers and derivatives for the production of cellulosic biopolymeric materials is focused on integration of these forms into biopolymer composites and composite films, as both fillers and polymer matrices. A biopolymer composite material is usually defined as a combination of two or more different components, in which one or more phase(s) is bio-based or biodegradable, and in which one acts as a filler or reinforcer, while the other provides a resin or polymer matrix.^72^ The blending of two or more biopolymers can produce new biopolymers designed for specific requirements. The properties of a green composite cannot be obtained by using any of the components on its own. Biopolymer composite properties depend on several factors, including the properties of the matrix, properties and aspect ratio of the reinforcing fibers, amount of fibers in the composite, geometry and orientation of the fibers in the composite, and fiber/matrix interfacial adhesion. Biocomposites can be categorized into three types: partially biodegradable, in which only the biofiller phase is biodegradable; completely biodegradable, in which the matrix is biodegradable; and hybrid biocomposites, which consist of two or more biofibers combined with a polymer matrix.^73^ Recent developments in biocomposite research has enabled a transition from petroleum-based polymers, *e.g.*, PE and PP, to naturally derived biopolymers, *e.g.*, based on cellulose and starch, and substituting glass fibers by cellulose fibers. Cellulose-based biocomposite systems are manufactured from cellulose, which can function as both a reinforcer and a matrix (host material).

### 5.1. Polymer composite materials with natural cellulose fibers and cellulose derivatives as filler

Natural cellulose fibers can be used for reinforcement. This cellulosic form has a number of useful properties such as low density, low cost, non-toxicity, renewability, recyclability, and good mechanical properties, making it attractive as a filler for composite material polymer matrices. Natural cellulose fibers can be used to reinforce biodegradable polymer composites^74^ and have been increasingly used as an alternative to talcum and glass fibers for plastic reinforcement. However, the strengths of natural fiber composites are lower than those of the glass fiber composites. This is because of the incompatibility between the generally hydrophobic host polymer matrix and hydrophilic natural fibers, and the lower thermal resistance of the cellulosic material. Furthermore, the mechanical properties of cellulose fibers deteriorate after moisture uptake. Structural modification of the cellulose fiber surfaces, *e.g.*, by chemical grafting and physicochemical treatments, is therefore necessary. Such modifications can improve the interfacial adhesion between the two phases; they can also improve the mechanical properties, biodurability, and weathering ability of the corresponding surface.^74,75^ However, a strength and stiffness equal to or surpassing those of glass fibers cannot be obtained with unmodified cellulosic fibers. Such high strength and stiffness have been achieved by using regenerated cellulose fibers as one of the cellulose derivatives and nanoscale cellulose particles. The data in Table 2 show that integration of cellulose lyocell fibers within a biopolymer matrix such as PLA and some films based on cellulose derivatives provides an alternative route to obtaining fully green polymer composites with good mechanical properties. Cellulose fibers and cellulose derivatives such as ethylcellulose can also successfully overcome the major obstacles to using PHA as an ideal biomaterial in a wide range of applications.^76–78^ These obstacles are related to a high degree of crystallinity, which results in the mechanical properties being unsuitable for biomedical and packaging applications, and poor thermal and barrier properties. The resulting composites have improved properties and are fully biodegradable, without adverse effects on their biocompatibility. Currently, the compatibility of cellulosic fillers and bioderived PHAs are attracting increasing attention. The latest research in this field has improved the compatibility of natural cellulosic fibers and PHA composite films by dip-coating, in which PHA is grafted using maleic anhydride to fabricate the composite and enhance its compatibility.^79^

**Table 2:** Effects of using cellulose fibers and their derivatives as filler or matrix in advanced polymer composites

Cellulose-based composites	Cellulose function in the composites	Resultant properties of cellulose interaction in the composites	Reference
Cellulose lyocell fibre/cellulose acetate butyrate composites	Matrix/filler	Increases tensile properties, dimensional stability, fibre and matrix compatibility, and biodegradability	85
Cellulose lyocell fiber/PLA	Filler	Unexpectedly high biodegradability, significantly high mechanical characteristics	86
(Ethyl)cellulose or (hydroxypropyl)cellulose/poly(acrylic acid) polymer composites with calcium phosphate-deposited	Filler	Increases thermal and mechanical performance	87
Cellulose fiber/polystyrene composites	Filler	Increases flexural storage modulus and the processing speed	88
Cellulose fiber/high-density polyethylene composites	Filler	Improves thermal and mechanical properties	89
Cellulose particles/chitosan composite film	Filler	Enhances mechanical properties and adsorption capacity of chitosan film	90
Regenerated cellulose film/bio Br composite	Matrix	Cellulose film provides a cavity for the BiOBr particles and enlarges the specific surface area through possessing a porous surface structure to exhibit efficient photocatalytic activity	91
Cellulose/MMT clay composite films	Matrix	High strength cellulose composite films with excellent antibacterial activities	92
Cellulose film/graphene oxide composite	Matrix	Superior mechanical performances and excellent ultraviolet-shielding properties	93
Cellulose acetate/hydroxyapatite mineral composites	Matrix	A useful application of the pollutants absorption resulted from uniform and good ductility of a cellulosic polymer, strong interaction existed between HAp and cellulosic polymer	94
Carboxymethyl cellulose/carbon fibers composites	Matrix	Cellulose gives the functional composites a great potential in sensing elements in paper electronics	95
Cellulose paper/carbon nanotube or regenerated cellulose, film/carbon nanotube composite	Matrix	The resulted composite is flexible, mechanical toughness, thermal stable, has uniform electrical conductivity, and suitable for biotechnological applications	96 and 97
Methylcellulose/keratin hydrolysate composite membranes	Matrix	The combines both properties of proteins and polysaccharides improves mechanical and thermal properties	98
Cellulose fibers/iodine composite	Matrix	Cellulose makes the composite with good conductor of photogenerated carriers and enhances the conductivity	99
Cellulose acetate membrane/polyaniline	Matrix	Cellulose derivative membrane enhances the conductivity and mechanical biocompatible properties	100
Polyhydroxybutyrate (PHB)/ethyl-cellulose composite film	Filler	Ethyl-cellulose reduces (PHB) crystallinity, promotes its degradation under physiological conditions and enhances physical barrier property without undue influence on biocompatibility. The resulted polymeric material being suitable for biomedical and coating aims	77 and 78
(PHB)/cellulose fibers composite	Filler	Cellulose fibers enhance physical–mechanical characteristics. The resulted polymeric material being suitable for the packaging industry	76

### 5.2. Polymer composites and composite films based on matrices of cellulose and its derivatives

Cellulose is usually considered to be a good candidate for the host material because it can improve the stability, maintain a special morphology, and control nanoparticle growth.^80^ Some semi-synthetic derivatives of cellulose provide important classes of chemically modified polymers. These derivatives have been extensively investigated to develop new biopolymeric materials with innovative physical and chemical properties.^81,82^ Cellulose esters and ethers are typical and common examples of chemically produced modified cellulose derivatives. In particular, cellulose derivatives such as cellulose acetate butyrate and cellulose acetate propionate are potential polymeric matrices for the production of sustainable, environmentally friendly, recyclable bio-based composites from renewable raw materials.^83,84^ Generally, when a hydrophilic and biocompatible matrix based on cellulose or its derivatives is combined with conducting electroactive materials such as metal ions and oxides, carbon nanotubes (CNTs), graphene and graphene oxide, conducting polymers, and ILs, through doping, blending, or coating, new functional composite materials such as electroconductive cellulose composites can be obtained. Such composites can provide a biocompatible interface for microelectronic devices, biocompatible energy scavenging, electrically stimulated drug-release devices, implantable biosensors, and neural prosthetics.^101^ For example, graphene oxide can be combined with a cellulose matrix to give a graphene oxide/cellulose composite. These interactions improve the electrical conductivity, and thermal and mechanical properties of the resulting composite films.^102^ A conducting polymer can also be coated or deposited on the surfaces and interiors of the pores in microporous mixed cellulose derivative membranes by various chemical oxidative polymerization techniques. The obtained composites have applications as conductors, biosensors, and electromechanical devices.^100,103^ An important class of advanced cellulose polymeric materials was obtained by use of unmodified cellulose as a matrix reinforced with inorganic nanoparticles to give hybrid inorganic–organic nanocomposites. Polymer matrix nanocomposites and hybrid nanocomposites based on natural cellulose are a new class of functional nanomaterials with improved conductive, optical, thermal, and mechanical properties.^104^ For example, natural cellulose-based composites containing copper show rapid and efficient inhibition of a multi-drug resistant wound pathogen, at a conductivity of 0.15 S cm^−1^.^105^

The use of cellulose derivatives in organic–inorganic superabsorbent composites is attractive and promising because they give excellent performances and are environmentally friendly.^106^ Recently, composite materials based on cellulose derivatives have been used instead of petroleum-based plastics in biodegradable polymer films. For example, a cellulose derivative was used as the host face in biopolymer-based electrolyte films, which have great potential for use in electrochemical devices such as proton batteries and solar cells.^27^ Water-soluble carboxymethylcellulose and hydroxyethylcellulose have attracted much interest because of their non-toxicity, good biocompatibility, high viscosity, transparency, and good film-forming ability.^107^ Biodegradable composite materials based on aliphatic polyesters and cellulose derivatives are among the most effective composites in reducing environmental stress; they perform well, are cheap, and have good biocompatibility.^108^ The effects of using cellulose fibers and their derivatives as fillers or matrices in advanced polymer composites are listed in Table 2.

When cellulose and its derivatives function as a matrix, the obtained composites have good uniform electrical conductivity and great potential as functional materials. These functional materials have important applications in electrochemical and energy-saving devices. A biodegradable CNT/cellulose paper composite has provided a novel and simple method for creating multifunctional biomaterials for electronic, magnetic, semiconducting, biosensing, and biotechnological applications. Such promising multifunctional composites have low composite phase costs, and excellent mechanical and thermal properties. In addition, they enable introduction of uniform electrical conductivities. Moreover, a simple and effective method for the preparation of CNT/cellulose papers or films has recently become available.^96,97^ This cellulosic biodegradable composite is comparable to multifunctional CNT/epoxy non-biodegradable composites, and has similar technological applications in various fields such as aerospace, automobiles, fuel cells, and electromagnetic sensors.^109^

## 6. Advances in biopolymeric materials based on nanoscale cellulosic materials as third route for cellulosic biopolymer production

**Fig. 4:**
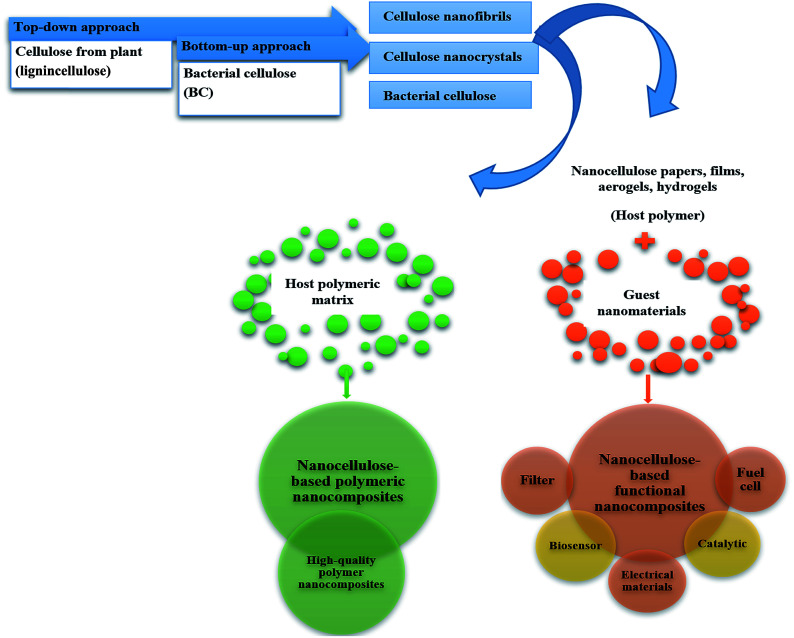
Schematic diagram of potential applications of nanocellulose-based polymeric nanocomposites depending on role of cellulose.

In current use of nanocellulose in biopolymer production, nanocellulose can be integrated into two types of polymer nanocomposites: nanocellulose-based nanopolymer composites and nanocellulose platform-based nanocomposites (Fig. 4).

### 6.1. Nanocellulose-reinforced polymers: nanocellulose-based bio- and nano-polymer nanocomposites

Polymer nanocomposites have gained much attention because of their low environmental pollution, low safety risks, renewability, and potential for low-cost industrial-scale production.^110^ Cellulosic nanomaterials have potential applications as the next generation of renewable reinforcements for the production of high-performance biocomposites^111^ because of their advantages such as nanoscale dimensions, high aspect ratios, low densities, low manufacturing costs, and good biodegradability. Their excellent mechanical properties are also imparted to the corresponding nanocomposites.^74,112,113^

The high crystallinity and possibility of exploiting the high stiffness and strength of cellulose crystals in composite applications are the basis of nanocellulose use for reinforcement.^111^ There are various types of nanocellulose, including microfibrillated cellulose, nanofibrillated cellulose (NFC), nanocrystalline cellulose (NCC) or CNCs, and bacterially produced cellulose or BC.^114^ These types of nanocellulose can be converted to various reinforcing structures for distributed reinforcement, planar reinforcement, or continuous networked structures. Nanocellulose has been used to reinforce various polymers, including PP, polystyrene, and high-density PE, since 1987; this was its first use in nanocomposites.^115^ The incorporation of small amounts of nanometer-sized fillers gives composites with the enhanced properties needed for many industrial and technological applications.^116^

A low loading of non-acid-hydrolyzed nanocellulose (NFC and BC) can yield composites with mechanical properties that are better than those of the neat polymer, just in the case of the elastomeric state of the matrix polymer. However, for other polymer matrices, high-performance nanocellulose (NFC and BC)-reinforced structural polymer composites are obtained when the nanocellulose loading is increased beyond 30 vol%.^111^

A key challenge is nanocellulose dispersion in hydrophobic polymer matrices, therefore methods for obtaining a uniform distribution of nanocellulose within nanocomposites are needed. The surface has to be engineered to achieve good dispersion levels and good bonding of the filler within the polymer matrix, which is basically hydrophilic. CNCs are considered to be ideal for nano-reinforcement of polymer matrices because of the abundant hydroxyl groups on their surfaces and their high surface-to-volume ratios, which make them suitable for many types of surface functionalization with various chemicals.^117^ There are various chemical surface modification methods for CNCs, such as esterification, etherification, oxidation, silylation,^118,119^ and polymer grafting on the nanocrystal surfaces.^120^ Such chemical surface modification methods facilitate the incorporation and dispersion of CNCs in hydrophobic polymer matrices.^112^

When surface-modified highly crystalline CNCs are used for reinforcement of polymer matrices, even at low concentrations, the resultant nanocomposites have better mechanical, thermal, and barrier properties than the unfilled polymer matrix.^121,122^

Nanocellulose is the best choice for improving the biodegradability of various composites. Life cycle assessment was used to evaluate the environmental impacts of epoxy composites reinforced with BC and NFC.^123^ A comparison with neat polylactide (PLA) and 30 wt% randomly oriented glass–fiber-reinforced PP composites showed that nanocellulose-reinforced epoxy composites with high nanocellulose loadings are greener than the best-performing commercially available bioderived polymers. The interactions of various nanoscale cellulose fibers with the matrix and the resulting reinforcing effects on the matrix polymer are listed in Table 3. It should be mentioned that the use of nanocellulose as a nanocomposite filler enables lower amounts of non-renewable materials to be used in the polymer matrix. When the host polymer matrix is also obtained from a renewable source, *e.g.*, PLA with nanocellulose, the resulting nanocomposite consists entirely of renewable components^124^ and will be completely renewable and biodegradable. The presence of cellulose as a filler in such composites maintains the biodegradability of the PLA matrix, which is often inferior to those of traditional petroleum-based polymers.^125^ Use of a nanocellulose filler also addresses the property-performance (thermomechanical performance) gap between this renewable polymer and petroleum-based polymers, as shown in Table 3. Fully renewable and biodegradable polymer composites of PLA with low nanocellulose loadings (2.5–6 wt%), especially of surface-modified CNCs and BC, are the most promising polymer composites for industrial use because of their compatibility, improved rheological, thermal, and mechanical properties, high biodegradability, non-toxicity, and completely renewability. Both the composite phases, *i.e.*, the PLA matrix and the CNC nanofibers, are forms of cellulose, obtained by cellulose deconstruction and reformation, respectively. This type of polymer composite is a viable alternative to important petroleum-based polymers such as poly(ethylene phthalate). It also offers biodegradable packaging materials, including food packaging,^126^ with low raw material costs because several facilities for large-scale production of CNCs have been announced in the last few years. The advantages of such bionanocomposites enable them to compete directly with mainstream petroleum-based plastics such as PE, PP polystyrene, and poly(vinyl chloride). However, the compatibility of nanocellulose with the PLA matrix is still a key factor. Several methods for increasing the compatibility of nanoparticles with the matrix have recently emerged.^146,151^ One of the most recent studies in this field showed that the incorporation of CNCs into electrospun nanofibers of poly(vinyl alcohol)/CNC improves the dispersion of cellulose nanowhiskers (CNCs) in the PLA matrices.^152^ However, more research is still needed to make nanocellulose dispersion in, and reinforcement of, PLA and other polymer matrices commercially effective. Such use of cellulose in polymeric materials gives better results than similar use of nanocellulose for reinforcing biopolymers such as starch. Such polymer composites have enhanced thermomechanical properties, strong interfacial adhesion, and potential applications in food packaging. Prakobna *et al.*^153^ used bioinspired core–shell CNFs to enhance the moisture stability of this type of composite; this is a major challenge with regard to biocomposites. However, starch needs to be plasticized and the fundamental properties such as mechanical properties and moisture sensitivity of plasticized starch-based materials need further improvement. Nanocellulose can also be used as an excellent green filler to enhance the properties of PHAs, opening the door for high-quality, PHAs/nanocellulose nanocomposites with a particular tailored target application in the food sector. Such nanocomposites have helped to overcome the most critical limiting factors of PHAs properties towards wide-scale application, especially in the food industry as unique biodegradable food packaging.^148,154^ Toward commercial application of PHAs, particularly in the food packaging, the lack of adequate flexibility, thermal stability and barrier properties for the water vapour, oxygen, and flavourful compounds are the main significant problems.^149,155^ The incorporation of nanofillers into PHAs as a solution for this limitation has been reported in many studies.^155–157^ However, this incorporation of nanofillers may result in a potential toxicological migration of degradation products produced during either processing or biodegradation from the nanofillers to the food. Also, a reduced rate of biodegradation of PHAs has been reported by increasing nanoparticle content.^157–159^ In contrast, the incorporation of nanoscale cellulosic forms such as CNCs, CNWs, and BC into a matrix consisting of PHA or its PHBV copolymer can improve their properties (*e.g.*, the physical, barrier, thermal, mechanical, rheological, hydrophilic, and crystallization properties of the biopolymer),^147,160^ while ensuring that their fully biodegradable and non-toxic nature is retained.^154^

**Table 3:** Recent trends in nanocellulosic polymeric materials, showing reinforcing effects of different cellulose nanoparticles in various polymer matrices on nanocomposite performance

Polymer nanocomposites reinforced with nanocellulose	Cellulose nanofillers addition effect on the polymeric nanocomposites properties	Loading (%)	Reference
MCCS/PLA matrix and organophilic silica	Increases crystallinity degree and tensile modulus in the resulted nanocomposites	3 wt%	127
BC nanopapers/polylactide	Increases mechanical properties	65 vol%	128
CNCS/polymer (IPN) hydrogels	Substantial improvements in the mechanical properties	50 wt%	129
CNCS/polyurethane	Enhances thermo-mechanical properties	30 wt%	130
CNFS/poly(vinyl acetate)	Improves water resistance and mechanical performance	10 wt%	131
Cellulose whiskers/natural rubber	Increases thermal stability	10 wt%	132
CNFS/polyvinyl alcohol	Increases mechanical properties	Up to 40 wt%	133
CNFS/poly(ε-caprolactone) CNFS/polycaprolactone/polypropylene	Increases surface wettability, mechanical, and thermal properties	1 wt%	134 and 135
CNCS/polypropylene	Increases mechanical properties	2 wt%	136
Methylcellulose/CNCS	Improves mechanical and barrier properties of the films	8 wt%	137
NCCS/chitosan/polyvinyl alcohol	Improves barrier properties	5 wt% to 15 wt%	138
Acetylated bacterial cellulose/poly(lactic acid)	Increases thermal and mechanical properties. Surface acetylation of the BCs increases their compatibility with the PLA matrix	Up to 6 wt%	139
Lignin-coated CNCS/poly(lactic acid) (PLA)	Improves rheological and thermo-mechanical properties. Excellent dispersion and compatibility of L-CNCs with PLA	0.3 wt% to 0.5 wt%	140
Modified CNCS/epoxy	Enhances thermo-mechanical properties	0.5 wt%	141
BC/plasticized starch with plasticizer and crosslinked with citric acid	Substantially enhances thermo-mechanical properties, exhibits a strong interfacial adhesion, and resulted composite shows potential to further applications of packaging	60 wt%	142 and 143
CNCS/cellulosic paper with starch	Improves thermal and mechanical properties	0.3 wt%	144
CNCS/g-rubber/PLA, CNCS/PLA through noncovalent modification with PLLA-based surfactants, and spherical nanocellulose formats (SCNFs)/PLA	CNCS greatly improve tensile toughness, barrier, thermal properties, and the resulted composites exhibit highly biodegradable and show potential to replace poly(ethylene terephthalate). Surface modification clearly increases the compatibility of the nanoparticles with the matrix	2.5 wt% and 5 wt%	145 and 146
Cellulose nanowhiskers (CNWs), BC nanowhiskers (BCNW) or CNC/polyhydroxyalkanoates (PHA)	Nanocellulose presents a nucleating effect on the PHA matrices and increases their thermal stability. Also, nanocellulose improves barrier and mechanical properties at low nanofillers loadings and low relative humidity with good compatibility. Resulted composites show potential applications of food packaging	1 wt% to 3 wt%	147–149
A novel PLA-PHB blends/CNCs	CNCs increase the crystallinity, improve the processability and increase the interfacial adhesion in the systems. Furthermore, the migration levels for these films were also well below the European legislative limits required for their use as food packaging materials showed a new perspective for their industrial application as food packaging	5 wt%	150

However, several disadvantages limit their competition with traditional synthetic plastics. The most serious disadvantages are their low compatibility with highly hydrophilic nanocellulose, especially BC, the hydrophobic nature of microbial PHA, and the high production costs of PHAs. Several advances have been made in these areas. Bhardwaj and coworkers^154^ reported a cost-effective technique for the dispersion of CNCs in PHB through a solvent exchange/casting method. Martínez-Sanz *et al.*^161^ achieved high dispersion of BC nanowhisker nanofillers in waste-derived PHAs. Arrieta *et al.*^150^ achieved another promising multifunctional film in this area. In this nanocomposite, the CNCs were added into (PLA-PHB) blends to increase the interfacial adhesion in the systems maintaining the thermal stability.

However, nanocellulose-reinforced polymeric materials still need to be improved for them to be truly competitive with traditional petroleum-based plastics, especially in terms of achieving full compatibility of the nanoparticles with the matrix. Full compatibility is still the most important target of future research in this area.

### 6.2. Nanocellulose as matrix platform in polymeric nanocomposite materials and their emerging applications

When nanocellulose is interwoven by bottom-up assembly, it can give a high-specific-surface-area, highly porous, and mechanically strong platform for a range of guest nanomaterials. These nanomaterials can be incorporated into the nanocellulose substrate *via* three methods, namely direct coating onto the nanocellulose surface, direct addition by nanocellulose dispersion, and formation of the guest nanomaterial in nanocellulose-based materials such as BC membranes. The resultant nanocellulose-based nanocomposites combine the advantages of the guest nanomaterial and the nanocellulose substrate and often show synergetic properties. In addition, they have many potential uses, including as antimicrobial filters, pollutant biosensors, catalytic activities, and sustainable energy devices.^162^Table 4 summarizes some recently developed nanocellulose-based nanocomposites and their potential applications.

**Table 4:** Summary of most important recent applications of nanocellulosic polymer matrices in various nanocomposite polymeric materials

Nanocellulose-based nanocomposites	Application	Reference
Nanocellulose/TiO_2_	Catalytic activity	175
Nanocellulose/Pd NPs	Catalytic activity	162
Surface functionalized BC/Au NPs	Catalytic activity	176
Nanocellulose aerogels/methyl aluminoxane	Catalytic activity	177
Nanocellulose/ZnO	Catalytic activity	162
BC/Pt NPs	Fuel cell	178
BC/carboxylic multi-walled carbon nanotubes	EBFCs	166
TOCNs^a^/carbon nanotube	Electrical materials	179
CMFS/tin-doped indium oxide thin layer	Solar cell	180
CNF_S_ paper/silver nanowires thin layer	Solar cell	175
CMFS/graphite, SiO_2_, LiFePO_4_	Li-ion battery	181
BC/SiO_2_	Li-ion battery	182
BC/graphene oxide	Li–S batteries	183
BC/supported CoFe_2_O_4_	Metal–air batteries	184
Nanocellulose/Au NPs	Excellent biosensors	162
CNC_S_/Ag NPs	Biosensors	185
PDDA^a^–CNC_S_/Au NPs	Biosensors	186
BC/Ag NPs	Antimicrobial activity	34
MCFS or CNFS/ZnO NPs	Antimicrobial activity	187

a PDDA: poly(diallyldimethyl ammonium chloride), TOCNs: (TEMPO)-oxidized cellulose nanofibrils.

In terms of the use of nanocellulose in catalytic nanocomposites, the nanocellulose typically acts as a catalyst support to hinder nanoparticle aggregation. There are mainly two categories of guest nanoparticles (NPs) used for catalytic applications including photocatalysts such as titanium dioxide nanoparticles TiO_2_ and precious metals such as Au, Ag, and Pt.^162^ Another area in which bulk nanocellulose/guest antimicrobial nanomaterials have performed well is air and drinking water purification; this is because they combine high removal efficiency with anti-fouling properties, which endows them with superior antimicrobial properties. The high surface : volume ratio of nanocellulose enables it to house larger amounts of antimicrobial nanomaterials such as Ag nanoparticles, to inhibit biofilm growth and prolong the filter life;^163^ it can also be used in biosensors, catalysts, and sustainable energy devices.^162^

One of the most important uses of nanocomposites based on nanocellulosic polymer matrices is in energy applications. Such applications have helped to meet the rapidly increasing demands for renewable material-based energy devices in recent decades.^164^ This is because of the excellent physical properties of nanocelluosic materials. Nanocellulose has also been used as a novel substrate for fuel cell fabrication and shows promise in energy applications.^162^ The hydroxyl groups on the nanocellulose backbone provide it with high hydrophilicity, which is crucial for the operation of polymer electrolyte membrane fuel cells.^165^ Recently, enzyme biological fuel cells (EBFCs)^166^ have been investigated as new green renewable energy devices that do not generate harmful intermediates and side products. EBFCs have drawn much attention because they can harvest electricity from renewable and abundant sources by using enzymes as catalysts for the oxidation of biofuels (most commonly glucose) and reduction of oxidizers (most commonly oxygen).^167^ Solar cells, sustainable organic batteries, and advanced three-dimensional networks of cellulose-based energy-storage devices^168^ are important energy devices based on nanocellulose nanocomposites.

Biocomposites made from nanocellulose-based biodegradable matrices represent a vital future alternative route to green nanocomposites, especially for use in bioenergy-storage and electrochemical devices. Most nanocomposite-based nanocelluloses do not have the mechanical and electronic properties that can be obtained by good nanofiller dispersion in composite polymer matrix materials. However, the use of nanocellulose nanopapers and aerogels can overcome this challenge and provide excellent, promising polymer matrices for dispersion of CNTs, which are the best electrode materials, to give well-mixed structures, with dispersion limits exceeding 40 wt%. These can be obtained by an assisted aqueous method, in which the aqueous medium forms long-term stable colloidal dispersions without the need for chemical functionalization of the CNTs or use of a surfactant.^169^ Simple, low-cost, sustainable alternatives for advanced strong functional composites with high electrical conductivity can be obtained from NFC/single-walled CNT and multiwalled CNT dispersions.^170,171^ In addition, promising, low-cost, strong functional composites with high electrical conductivity can also be obtained *via* existing simple methods for synthesis of nanocomposites from BC papers and ion gels with carboxylic multiwalled CNTs.^166,172^ The introduction of BC as a matrix for flexible energy-storage devices has high potential because BC is cheap,^173^ and has higher mechanical strength and better chemical stability than regular papers.^174^ Nanocellulose papers and gels/CNT nanocomposites offer biosustainable alternatives for advanced electrochemical and biofuel applications such as enzymatic EBFCs, new green energy devices, batteries, supercapacitors, and photovoltaics.

## 7. Concluding remarks and outlook

Various forms of cellulose are promising resources for biopolymer production *via* three major routes. The first route involves deconstruction of cellulose to polymerizable monomers. The second and third routes involve incorporation of different forms of cellulose, including natural fibers, cellulose derivatives, and nanocellulose, as fillers or matrices in polymer composites. Each route leads to various biopolymeric materials, making cellulose a dominant biopolymer feedstock. The effective use of cellulose will reduce the consumption of limited fossil resources.

Cellulose-derived monomers provide various types of sustainable biopolymers and functional biopolymeric materials, including classic, analogous, and novel polymers. Cellulose-based polymers therefore provide alternatives to oil-based polymers in numerous fields. Cellulosic forms, including natural fibers, nanocellulose, or cellulose derivatives, provide various types of promising polymeric materials. Incorporation of these materials into composite polymeric materials as fillers improves the composite properties. Their use as matrices gives multifunctional biopolymeric materials that have advanced biotechnological applications. Some of the recent advances in biotechnology have enabled the production of promising biopolymers from different cellulosic forms. In recent years, significant advances have been made, especially in terms of the cost, compatibility, characteristics, and performances of such polymeric materials. The most important cellulose-based biopolymers can be grouped into the following four types. (1) PLA biopolymer matrices, especially when they are filled with nanocellulose, give highly biodegradable tough, non-toxic, nanocomposites with good thermal and mechanical properties. This type of composite is based completely on different cellulosic forms, in two phases. This type of composite has widened the range of industrial applications of cellulosic bioplastics and enabled them to compete directly with many mainstream petroleum-based plastics. Types (2) and (3) consist of polymer matrices based on cellulose, cellulose derivatives, and nanocellulose papers, aerogels, and films reinforced with CNTs. They are simple, cheap, and strong functional composites with well-mixed structures, and high electrical conductivity. These composites are biosustainable alternatives for use in advanced electrochemical and energy-storage devices. (4) Incorporation of cellulose in its different forms (cellulose fibers, cellulose derivatives, and nanocellulose) has widened the use of PHAs, to give important biopolymeric materials. These materials overcome the main difficulties associated with their large-scale applications, namely their properties and cost. Cellulose-based LevA offers an inexpensive and renewable carbon source as a useful 3HV-related precursor for production of PHA at high concentrations; this significantly reduces the cost. The integration of different cellulosic forms as a filler into a PHA matrix provides fully green composites with improved properties and great potential as replacements for petroleum-based polymers for food packaging, everyday plastics, and medical applications. Cellulose is a crucial biopolymer resource. The highlighted routes for production of cellulose-based polymers will provide our society with appropriate polymeric materials. This will be helpful in the development of sustainable polymeric products to meet economic, environmental, and social needs. However, the substitution of petroleum-based polymers by cellulosic biopolymers is currently limited by challenges that need to be addressed in the coming years. These challenges include creating easy, low-cost, and less time-consuming methods for all stages of cellulosic biopolymer production, including cellulose isolation from lignocelluloses, synthesis of appropriate forms of cellulose, and polymerization. In addition, methods for making the two phases of polymer composites fully compatible need to be developed for many cellulose-based composite materials. Solutions to these challenging problems require many different approaches, based on chemistry, material science, and process engineering. This critical review will help researchers in the planning, selection, and development of various forms of cellulose for a range of polymeric material applications.

## Conflicts of interest

There are no conflicts to declare.

## Supplementary Material
